# Neighborhood Quality and Attachment: Validation of the Revised Residential Environment Assessment Tool

**DOI:** 10.1177/0013916516634403

**Published:** 2016-07-27

**Authors:** Wouter Poortinga, Tatiana Calve, Nikki Jones, Simon Lannon, Tabitha Rees, Sarah E. Rodgers, Ronan A. Lyons, Rhodri Johnson

**Affiliations:** 1 Cardiff University, Wales, UK; 2 Swansea University, Wales, UK

**Keywords:** neighborhood quality, place attachment, audit tool, validation, inter-rater reliability

## Abstract

Various studies have shown that neighborhood quality is linked to neighborhood attachment and satisfaction. However, most have relied upon residents’ own perceptions rather than independent observations of the neighborhood environment. This study examines the reliability and validity of the revised Residential Environment Assessment Tool (REAT 2.0), an audit instrument covering both public and private spaces of the neighborhood environment. The research shows that REAT 2.0 is a reliable, easy-to-use instrument and that most underlying constructs can be validated against residents’ own neighborhood perceptions. The convergent validity of the instrument, which was tested against digital map data, can be improved for a number of miscellaneous urban form items. The research further found that neighborhood attachment was significantly associated with the overall REAT 2.0 score. This association can mainly be attributed to the property-level neighborhood quality and natural elements components. The research demonstrates the importance of private spaces in the outlook of the neighborhood environment.

## Introduction

### Neighborhood Quality and Attachment

An impressive body of literature has accumulated over past three decades on place attachment and related concepts, such as sense of place and place identity. This research has provided important insights into how individuals develop and experience bonds with their physical surroundings (Low & Altman, 1992; Manzo & Devine-Wright, 2013). A recent review describes a dynamic field of research that has developed over the years, with a wide variety of definitions and theoretical frameworks, as well as empirical studies on the origins, development, and outcomes of people’s emotional bonds with places (Lewicka, 2011).

The current study concerns the role of the quality of the neighborhood environment in the emotional bonds residents have with their neighborhood. It builds upon work that identified a range of physical environmental features that can directly or indirectly predict place attachment (e.g., Bonaiuto & Alves, 2012; Bonaiuto, Fornara, & Bonnes, 2003; Brown, Perkins, & Brown, 2004; Hidalgo & Hernandez, 2001; Hur & Morrow-Jones, 2008; Stedman, 2003). Previous research has shown that the *presence of green or quiet areas* (e.g., Bonaiuto, Aiello, Perugini, Bonnes, & Ercolani, 1999; Kim & Kaplan, 2004; Pacione, 1986) and *absence of incivilities* (e.g., litter, graffiti, vandalism; Brown, Perkins, & Brown, 2003; Brown et al., 2004) are particularly important for neighborhood attachment. There are good reasons for people to develop positive bonds with such environments: Natural elements provide opportunities for mental restoration and recreation (Coombes, Jones, & Hillsdon, 2010; Hartig, Mang, & Evans, 1991; Kaplan, 1995; Lee & Maheswaran, 2011), and an absence of incivilities and other signs of decline engenders feelings of security and safety (LaGrange, Ferraro, & Supancic, 1992; Wilson & Kelling, 1982). Incivilities may act as a social indicator of poorly functioning neighborhoods and, as a result, trigger further disorder and petty criminal behavior (cf. Keizer, Lindenberg, & Steg, 2008). In addition, urban form features, such as natural surveillability and physical and symbolic boundaries, afford feelings of safety and social interaction in line with the principles of defensible space and CPTED (crime prevention through environmental design; Abdullah, Hedayati Marzbali, & Maghsoodi Tilaki, 2013; Newman, 1972; Skjaeveland & Garling, 1997; Taylor, Gottfredson, & Brower, 1984).

Most of the research on neighborhood quality and attachment has thus far relied upon residents’ own perceptions rather than independent observations (Lewicka, 2011). This is problematic because neighborhood perceptions cannot be separated from the emotional bonds people have or develop with their neighborhood. Residents with higher levels of attachment or place identity are less likely to acknowledge existing environmental pollution (Bonaiuto, Breakwell, & Cano, 1996) and more resistant to changes in the physical environment (e.g., Devine-Wright, 2009). If measures of neighborhood quality and attachment are collected with the same instrument, common method variance (or same-source bias) can never be ruled out as an explanation for an observed relationship (Podsakoff, MacKenzie, Lee, & Podsakoff, 2003). Common method variance can be avoided by making independent neighborhood observations using neighborhood audit tools.

### Neighborhood Audit Tools

Neighborhood audit tools have several advantages over other forms of data collection, although they are not strictly “objective” in the sense that they are still subject to observer bias. They avoid problems with common method variance and are able to capture a wider range of factors than are available from routine data sources (Schaefer-McDaniel, Caughy, O’Campo, & Gearey, 2010; Nickelson, Wang, Mitchell, Hendricks, & Paschal, 2013). Their use has therefore become increasingly common in environmental psychology (e.g., Abdullah et al., 2013), urban studies (e.g., Clifton, Livi Smith, & Rodriguez, 2007), and public health research (e.g., Schaefer-McDaniel et al., 2010).

A large number of different neighborhood assessment tools have been developed over the past two decades (see, for example, Nickelson et al., 2013, for a recent systematic overview). These tools have been developed with different purposes in mind. Many of the audit instruments focus on urban form factors, such as land-use patterns, access to amenities, traffic safety, aesthetics, and (street/path) maintenance, that may influence participation in physical activity (Brownson, Hoehner, Day, Forsyth, & Sallis, 2009; Evenson et al., 2009; Millington et al., 2009; Pikora, Giles-Corti, Bull, Jamrozik, & Donovan, 2003). Other instruments have been designed to assess the overall quality of the neighborhood environment to better understand the role of environmental factors in public health (Araya et al., 2007; Dunstan, Fone, Glickman, & Palmer, 2013; Dunstan et al., 2005; Weich et al., 2002).

The Built Environment Site Survey Checklist (BESSC), Pregnancy, Infection, and Nutrition (PIN3) observation tool, Residential Environment Assessment Tool (REAT), and Built Environment Assessment Tool (BEAT) are all examples of public health instruments that were developed by different research groups. The revised Residential Environment Assessment Tool (REAT 2.0) that is used in the current study is based on the original REAT tool developed in 2001.

The BESSC, developed in the United Kingdom by Weich and colleagues (2002), covers built form and housing factors as well as aspects relating to neighborhood quality, incivilities, and territorial functioning. The PIN3 observation tool was primarily developed to better understand salient physical environment features that may enhance outdoor physical activity (Evenson et al., 2009) and contains the same physical incivilities, territoriality, and social spaces elements included in other neighborhood quality audit tools (e.g., Caughy, O’Campo, & Patterson, 2001). The REAT was designed to provide a contextual measure of the overall quality of the residential environment based on observed incivilities, territorial functioning, defensible space, and natural elements (Dunstan et al., 2005). The BEAT, developed by Araya et al. (2007) in a Latin American context but part-based on the REAT instrument, identified four factors to assess the built environment: (a) general neighborhood quality; (b) facilities, noise, and traffic in the area; (c) public green areas; and (d) empty buildings/sites.

Although a number of audit instruments have been developed to assess the overall quality of the neighborhood environment, there are a number of issues that need to be addressed to improve their utility in studying human–environment interactions and relationships. First, it is critical that an audit instrument is valid and reliable (Craik & Feimer, 1987). Although many tools have been shown to be reliable (e.g., Dunstan et al., 2005; Foster, Giles-Corti, & Knuiman, 2011; Paquet, Cargo, Kestens, & Daniel, 2010; Weich et al., 2001), very few of them have been systematically validated to test whether they capture the appropriate content and converge with other methods of measurement (Brownson et al., 2009). Second, neighborhood quality assessments have been linked to only a limited number of health outcomes. Given the importance of neighborhood quality for the development of people’s emotional bonds with places, the predictive or criterion validity should be assessed with a wider range of relevant psychological outcomes, such as residential satisfaction and attachment. Third, neighborhood quality instruments have thus far primarily focused on public elements. However, the urban environment is made up of both public and private spaces. The extent to which residents invest in their local environment may be equally important for the overall outlook of a neighborhood, in particular, impressions left by house maintenance and upkeep (Bonaiuto et al., 2003; Ng, Kam, & Pong, 2005; Perkins, Wandersman, Rich, & Taylor, 1993; Taylor et al., 1984; Wilson & Kelling, 1982). Work by Foster and colleagues (2011) has shown that house attributes that promote natural surveillance and house upkeep have the potential to discourage physical incivilities in suburban environments. The combined role of public and private aspects of neighborhood quality on residents’ quality of life and neighborhood attachment has, however, not been studied before.

### The Research

#### The REAT 2.0

In this article, we describe the validation of the REAT 2.0 to address the issues described above. The REAT 2.0 builds upon the original instrument that was developed in 2001 (Dunstan et al., 2005). The instrument was reviewed as part of a project on housing, neighborhoods, and health (Rodgers et al., 2014) to monitor improvements to the estate environment as part of a social housing regeneration program and to examine the role of neighborhood quality in producing longer term health benefits from the program. The instrument was developed further with the intention to (a) condense the data collection form, standardize rating scales, and devise more intuitive formatting and instructions; (b) make it more reliable by substituting new items for those that formerly had poor inter-rater reliability (IRR); (c) strengthen its theoretical underpinnings by considering more recent empirical evidence; and (d) increase its versatility by modularizing the tool.

**Figure 1.**
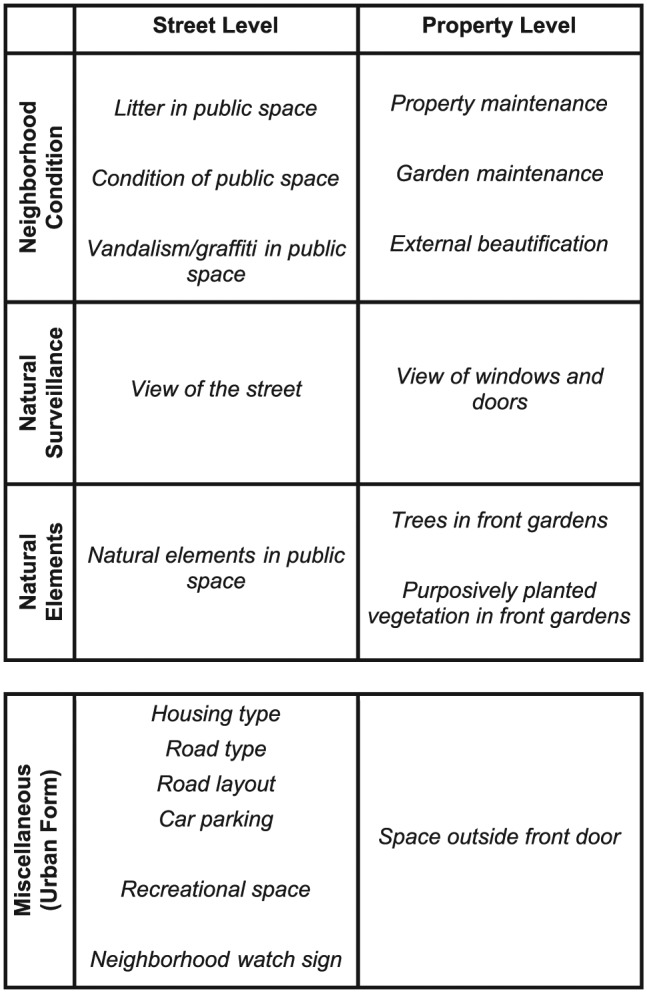
Structure of the REAT 2.0. *Note.* REAT 2.0 = Revised Residential Environment Assessment Tool.

The REAT 2.0 was shortened from 30 to 18 questions and restructured according to four separate dimensions (see Figure 1). Items from the original REAT instrument that were less reliable (e.g., condition of paths; Dunstan et al., 2005) or features that were observed only infrequently in previous studies (e.g., stray dogs roaming) were changed or removed from the instrument. Three of the REAT 2.0 dimensions (i.e., *neighborhood condition, natural surveillance*, and *natural elements*) are used to make an assessment of the overall quality of the neighborhood environment. The fourth *miscellaneous* dimension captures a number of urban form aspects that are not directly part of the overall neighborhood quality assessment but are used to characterize the neighborhoods under assessment. In contrast to other neighborhood audit tools, REAT 2.0 explicitly covers both public and private spaces of the neighborhood environment, that is, street- and property-level observations (see Figure 1). The neighborhood condition dimension was intended to capture the condition of both public and private spaces that make up the neighborhood environment (Perkins et al., 1993; cf. Taylor et al., 1984), the natural surveillance dimension to capture the elements of surveillance of the street and houses (cf. Newman, 1972), and the natural elements dimension to record “green” elements in both public (e.g., a park or tree-lined road) and private (e.g., purposefully planted vegetation in front gardens) gardens. The resulting six core components, covering the neighborhood condition, natural surveillance, and natural elements at both the street and property level, can be used separately or combined to form an overall neighborhood quality score (“overall REAT 2.0 score”). Online Appendix A provides a description of how the six core components and overall REAT 2.0 scores are calculated. Although the miscellaneous urban form items at both the street and property level are not included in the overall neighborhood quality score, they are part of the validation exercise presented in this article.

#### Aims of the research

The research had two interrelated aims: First, to validate the REAT 2.0 audit instrument, in particular in relation to the aspects of neighborhood condition, natural surveillance, and natural elements; and second, to explore the associations of overall neighborhood quality with residents’ attachment to their neighborhood. These aims were achieved by the following objectives: (a) determine the IRR of the instrument from a set of independent observations; (b) assess the convergent validity by comparing REAT 2.0 observations with other neighborhood assessments, including the PIN3 audit instrument (Evenson et al., 2009) and digital map analysis (DMA); (c) determine the construct validity of the instrument through a neighborhood perception survey; and (d) examine the links between the independent REAT 2.0 assessments and residents’ self-reported attachment to their neighborhood. The latter was used as a test of the predictive validity of the REAT 2.0 instrument. We conducted two studies to address the aims of the research: the *Carmarthenshire Homes Standard (CHS) Neighborhood Quality Study* and the *Cardiff Neighborhood Perception Study*.

## Method

### The CHS Neighborhood Quality Study

#### The study

Neighborhood quality data were collected as part of a project examining the health benefits of a major social housing regeneration program in Carmarthenshire, Wales. Two hundred seventy-one unit postcodes (a full postcode designates an area with a number of addresses or a single major delivery point, for example, CF10 3NB) were scored on foot by pairs of trained observers between May 30 and August 8, 2012. The observers were instructed to determine housing type, road type, and layout together (Items 1-3), and then to conduct the rest of the street-level (Items 4-11) and property-level (Items 12-18) assessments independently without comparing notes (see Online Appendix B). The time needed to conduct the REAT 2.0 assessments depended on the size of the postcode but took on average 16 min to complete (*SD* = 9 min). All observers received training prior to conducting the assessments and had access to a user manual containing instructions, operational definitions, and photographs illustrating different grading scales. The street-level items are used to make general assessments of the postcode under assessment, whereas the property-level items require the observers to count the number of properties exhibiting a certain feature. A description of how the six core components and overall REAT 2.0 scores are calculated based on these observations is provided in Online Appendix A.

The pairs of independent assessments of the unit postcodes were used to determine the IRR of the revised audit instrument (Objective 1). Cohen’s kappa (κ) statistic was used to determine the IRR of categorical items, Spearman’s rank correlation (ρ) to determine the IRR of ordinal items and non-normally distributed REAT 2.0 components, and Pearson’s correlation (*r*) to determine the IRR of the overall REAT 2.0 score and normally distributed REAT 2.0 components.

### The Cardiff Neighborhood Perception Study

#### The study

The Cardiff Neighborhood Perception Study was conducted in summer 2013 to determine the convergent, construct, and predictive validity of REAT 2.0 (Objectives 2, 3, and 4, respectively). In this study, 50 unit postcodes were randomly selected from the Cardiff City area. The postcodes were visited on foot by a trained pair of observers who independently rated the quality of the environment using both the REAT 2.0 and selected items from the PIN3 neighborhood audit instrument (Evenson et al., 2009). The neighborhood assessments were conducted between July 3 and August 1, 2013. A 39-item Neighborhood Perception Questionnaire was subsequently sent to all 1,160 residential addresses within the 50 unit postcodes, delivering a 24% response rate (*n* = 279).

#### The PIN3 neighborhood audit instrument

Convergent validity refers to the extent to which different instruments are able to measure the same construct. Both the REAT 2.0 and PIN3 audit instruments (see Evenson et al., 2009) contain items that assess different aspects of neighborhood quality.^1^ In this study, the street-level neighborhood condition component is validated with a PIN3-derived “neighborhood condition” scale (PIN3 Items 21 “general condition of public spaces,” 27 “amount of litter,” and 29 “amount of graffiti”; Cronbach’s α = .62) and the property-level neighborhood condition component with a “condition of residential units and grounds” scale (PIN3 Items 4 “overall condition of most residential units,” 5 “overall condition of resident-kept grounds,” and 8 “presence of some form of decoration”; Cronbach’s α = .74). The convergent validity of the two components was determined by Pearson’s correlation coefficient (*r*). A number of the miscellaneous urban form items (housing type, car parking, and space outside front door) were validated with comparable PIN3 items (types of residential housing, on-street parking, and presence of border; PIN3 Items 3, 42, and 8, respectively) by Cohen’s kappa (κ).

#### The Neighborhood Perception Questionnaire

Construct validity refers to the extent to which an instrument measures the intended theoretical construct(s). In this study, the construct validity of REAT 2.0 is assessed using the neighborhood quality survey that was sent to all addresses within the assessed unit postcodes. The questionnaire was designed to capture the six core components of REAT 2.0 (excluding the miscellaneous urban form items). All questions could be answered on a standard 5-point agree-disagree scale, with higher scores representing a greater presence of the construct. *Perceived neighborhood condition* was measured by asking residents to what extent they agreed or disagreed that a list of eight incivilities and maintenance issues are a problem in their neighborhood (vandalism, graffiti, litter and rubbish, burglaries, antisocial behavior, uneven or dangerous pavement, dog waste, and poorly maintained street furniture). The scale was adapted from Poortinga, Dunstan, and Fone (2008). The items were combined into an internally consistent scale (Cronbach’s α = .84). Perceived neighborhood condition was expected to be associated with the street-level neighborhood condition component of REAT 2.0. *Perceived house condition* was measured with the statements “people in this neighborhood take good care of their property” and “people in this neighborhood take good care of their garden.” The two items were combined into a reliable scale (Cronbach’s α = .90). Perceived house condition was expected to be associated with the property-level neighborhood condition component of REAT 2.0. *Perceived natural surveillance* was assessed by respondents’ agreement with three statements (“all front doors and windows of my property are clearly visible from the street,” “all front doors and windows of my neighbors’ properties are clearly visible from the street,” and “I have a clear view of the street from my property”). The three items formed an internally consistent scale (Cronbach’s α = .86). The perceived natural surveillance scale was expected to be associated with both the street-level and property-level natural surveillance components of REAT 2.0. A reliable *perceived natural elements* scale was created from the items “there are plenty of trees in my neighborhood,” “there is plenty of greenery in my neighborhood,” “there are plenty of safe spaces for children to play in my neighborhood,” and “there are plenty of parks/green spaces in my neighborhood” (Cronbach’s α = .88). The perceived natural elements scale was expected to be associated with both the street-level and property-level natural elements components of REAT. The items for the perceived house conditions, perceived natural surveillance, and perceived natural elements were newly designed for the research, as no existing scales were available. We used similar phrasings to those contained in the REAT 2.0 instrument (see Online Appendix B).

Predictive validity refers to the extent to which an instrument can predict the outcome based on a criterion measure. In this study, the criterion measure is neighborhood attachment. We expected that a better overall neighborhood quality results in higher levels of neighborhood attachment. Neighborhood attachment was determined by averaging residents’ agreement with four statements (“overall, I am attracted to living in this neighborhood”; “I feel like I belong to this neighborhood”; “given the opportunity, I would like to move out of this neighborhood” [reversed]; and “overall, I think this is a good place to bring up children”). Together, the four items formed an internally consistent scale (Cronbach’s α = .86). The items were adapted from Buckner’s (1988) social cohesion scale that were intended to measure the “attraction-to-neighborhood” concept and subsequently validated by Fone, Farewell, and Dunstan (2006).

Both the construct and predictive validity of the REAT 2.0 instrument were determined by conducting a series of multilevel regression models using the *MLwiN software package* (Rasbash, Steele, Browne, & Goldstein, 2012). The data set consisted of 279 respondents (Level 1) clustered within 50 unit postcodes (Level 2). On average, 5.58 responses per sampling point were received with a standard deviation of 3.70. The regression coefficients were estimated by Monte-Carlo simulations with 15,000 iterations. The regression parameters were estimated with and without adjusting for gender, age, and tenure status.

### DMA

The postcodes of both the CHS Neighborhood Quality Study and the Cardiff Neighborhood Perception Study (total *n* = 321) were combined and submitted to a DMA to determine the convergent validity of a number of the miscellaneous urban form elements of the REAT 2.0 instrument, that is, housing type, road type, road layout, car parking, and recreational space, as well as “view of street” (reflecting street-level natural surveillance) and natural elements in public space (reflecting street-level natural elements). DMA was used to assess the convergent validity of the “urban form” items because it can generate spatial metrics that contain information about urban form. By combining different spatial metrics, we may be able to derive urban form types without direct observation using an audit instrument.

Discriminant analysis was conducted to determine the extent to which nine DMA parameters can be used to correctly classify the urban form at the postcode level. Discriminant analysis is a statistical technique that can be used to describe or predict a categorical dependent variable (in this case, the urban form items) from a set of independent variables (Tabachnick & Fidell, 2001). It constructs a number of discriminant functions that describe the pattern of differences among the predictors. The overall Bartlett’s chi-square (χ^2^) shows whether the functions successfully discriminate between the different categories. The factor loadings on the discriminant functions show which predictors contribute the most to the explanation of the differences.^2^ We calculated the classification accuracy by comparing the predicted group membership based on the discriminant model with the known group membership. The accuracy relative to a classification based on chance only indicates the extent to which the discriminant models can improve the prediction of the urban form types.

DMA replicates the process of pattern recognition that humans use to understand the makeup of the urban environment by processing digital maps. The use of pattern recognition using digital maps was established by Barr, Barnsley, and Steel (2004) who used Ordnance Survey (OS) maps to infer urban land use and successfully identified areas with similar construction dates by considering street layout patterns. Further refinements were made by Lannon, Alexander, and Jones (2007) and Alexander, Lannon, and Linovski (2009), who used OS MasterMap™ to better describe topographic features (such as road tracks and paths, railways, and buildings) using layers of points, lines, and polygons describing urban objects.

The DMA was used to generate nine parameters describing the urban form of the 321 unit postcodes. First, dominant housing type was determined by combining two measures: the number of buildings in direct contact with other houses and the number of buildings within a group. This combination allowed housing type to be defined into (a) detached (single building not directly contacting another building), (b) semi-detached (contact with one other building as part of a group of two buildings), or (c) terraced housing (contact with one or more other buildings as part of a group of at least three buildings). Other parameters derived from the DMA were (d) road width, (e) average distance from building to the road edge, (f) average angle of the shortest line from a building to the road edge, and (g) the aspect ratio of the roads within a postcode. The aspect ratio of the roads within a postcode reflects the ratio of the two sides of the smallest box that can fit into the whole road of a postcode. The road-related parameters are mainly used to determine road type, car parking, and natural surveillance of a postcode. Road width is thought to determine road type, average distance from boundary to road edge to determine off-road parking, and aspect ratio to determine natural surveillance (i.e., the smaller the aspect ratio, the straighter the road, and vice versa). The final set of parameters, that is, the (h) average plot area connected to a building and (i) total area of natural environment within a postcode area, was used to determine natural elements within the postcode.

## Results

### IRR

Table 1 shows the IRRs of the individual REAT 2.0 items. There was a high level of agreement between observers. Seven of the eight individual street-level items had Cohen’s kappa higher than .9, with the vandalism/graffiti item returning a kappa of 1.0, representing a perfect agreement between observers. The lowest kappa was found for the presence of a neighborhood watch sign (.77). The Spearman rho values for the property-level items ranged from .79 to 1.0. The lowest values were for the “space outside front door” (.79) and property maintenance (.85), whereas the highest value was for the view of ground floor windows or doors from the street (1.0). The IRR of the urban form items of housing type, road type, and road layout were not determined, as they were assessed mutually by the pairs of observers or prefilled by the organizer of the assessments.

**Table 1.** IRRs of Individual REAT 2.0 Items.

Item number^a^	Item description	REAT 2.0 component	Scale	IRR	*p*
Street-level observations					
1	Housing type	Miscellaneous	1-5	—	—
2	Road type	Miscellaneous	1-3	—	—
3	Road layout	Miscellaneous	1-4	—	—
4	Car parking	Miscellaneous	1-5	κ = .98 (.01)	<.001
5	Recreational space	Miscellaneous	0-1	κ = .98 (.01)	<.001
6	View of street	Natural surveillance	0-1	κ = .99 (.01)	<.001
7	Natural elements in public space	Natural elements	0-5	κ = .91 (.02)	<.001
8	Litter in public space	Neighborhood condition	1-4	κ = .96 (.02)	<.001
9	Condition of public space	Neighborhood condition	1-4	κ = .97 (.02)	<.001
10	Vandalism/graffiti in public space	Neighborhood condition	1-4	κ = 1.00 (.00)	<.001
11	Neighborhood watch sign	Miscellaneous	0-1	κ = .77 (.10)	<.001
Property-level observations					
12	Space outside front door	Miscellaneous	1-5	ρ = .79	<.001
13	View of windows and doors	Natural surveillance	0-1	ρ = 1.00	<.001
14	Trees in front gardens	Natural elements	0-1	ρ = .94	<.001
15	Purposively planted vegetation in front gardens	Natural elements	0-1	ρ = .96	<.001
16	Property maintenance	Neighborhood condition	0-1	ρ = .85	<.001
17	Garden maintenance	Neighborhood condition	0-1	ρ = .90	<.001
18	External beautification	Neighborhood condition	0-1	ρ = .96	<.001

*Note.* REAT 2.0 = Revised Residential Environment Assessment Tool; IRR = inter-rater reliability; κ = Cohen’s kappa; ρ = Spearman’s correlation; Scale = number of categories or range of the scale. The Spearman correlation for Item 12 represents the average correlation for counts for the five categories.

a The numbers refer to the items of the full REAT 2.0 audit instrument (see Online Appendix B).

Table 2 shows the IRRs of the overall REAT 2.0 score and its six constituent components. The correlation between the overall REAT 2.0 scores of the two independent observers was .96. The six REAT 2.0 components had correlation coefficients of .97 or higher. The property-level defensible space component had a perfect Spearman’s correlation coefficient (1.00; also see Item 13 in Table 1). Table 2 further shows that the score differences between the observer pairs were negligible.

**Table 2.** IRRs of the REAT 2.0 Components.

Component code	Components description	Scale	*M* (*SD*) R1	*M* (*SD*) R1 − R2	IRR	*P*
C1	Neighborhood condition (SL)	0-3	2.00 (0.55)	0.01 (0.10)	*r* = .98	<.001
C2	Natural surveillance (SL)	0-1	0.63 (0.48)	0.00 (0.06)	ρ = .99	<.001
C3	Natural elements (SL)	0-1	0.29 (0.22)	0.00 (0.07)	ρ = .97	<.001
C4	Neighborhood condition (PL)	0-3	1.77 (0.45)	−0.03 (0.103)	*r* = .97	<.001
C5	Natural surveillance (PL)	0-1	0.82 (0.24)	0.00 (0.00)	ρ = 1.00	<.001
C6	Natural elements (PL)	0-1	0.35 (0.17)	−0.00 (0.04)	ρ = .97	<.001
	Overall REAT 2.0 score	0-10	5.85 (1.04)	−0.01 (0.18)	*r* = .99	<.001

*Note.* REAT 2.0 = Revised Residential Environment Assessment Tool; R1 = rater 1; R2 = rater 2; IRR = inter-rater reliability; SL = street level; *r* = Pearson correlation; PL = property level; ρ = Spearman’s correlation.

### Convergent Validity: PIN3 Neighborhood Audit Instrument

Table 3 shows the associations between the street-level and property-level neighborhood condition components of REAT 2.0 and the PIN3-derived “neighborhood condition” and “condition of residential units and grounds” scales, respectively. Whereas the street-level neighborhood condition component was highly correlated with the “neighborhood condition” scale (*r* = .81, *p* < .001), the property-level neighborhood condition component was only weakly associated with the “condition of residential units and grounds” scale (*r* = .38), although still significant at the 1% level. Table 3 further shows that the three urban form items could not be validated with the PIN3 neighborhood audit instrument: The kappa for the “space outside front door” item was moderate (κ = .58), and the kappas for “housing type” (κ = .10) and “car parking” (κ = .20) were low.

**Table 3.** Convergent Validity: Associations Between REAT 2.0 and PIN3 Components.

Component code or item number^a^	Component/item description	PIN3 component/item description	Association	*p*
C1	Neighborhood condition (SL)	Neighborhood condition	*r* = .81	<.001
C4	Neighborhood condition (PL)	Condition of residential units and grounds	*r* = .38	<.01
1	Housing type	Types of residential housing	κ = .10 (.08)	<.05
4	Car parking	On-street parking	κ = .20 (.12)	.055
12	Space outside front door	Presence of border	κ = .58 (.13)	<.001

*Note.* REAT 2.0 = Revised Residential Environment Assessment Tool; PIN = Pregnancy, Infection, and Nutrition; SL = street level; PL = property level; *r* = Pearson’s correlation; κ = Cohen’s kappa.

a The numbers refer to the items of the full REAT 2.0 audit instrument (see Online Appendix B).

### Convergent Validity: DMA

Table 4 shows the results of a series of discriminant analyses in which the categories of the five miscellaneous urban form items of the REAT 2.0 instrument as well as the street-level natural surveillance and natural elements components were predicted by a combination of the nine indicators generated through DMA. It appeared that housing type, car parking, and view of the street can be determined by DMA to a relatively high level of accuracy.

**Table 4.** Convergent Validity: Model Statistics of Discriminant Analyses Predicting REAT 2.0 Items From DMA-Derived Urban Form Indicators.

Item number^a^	Item description	Explained variance (%)	χ^2^ (*df*)	*p*	Classification accuracy (%)	
					Chance	Discriminant model
1.	Housing type	71.9	392.120 (36)	<.001	30.2	69.4
2.	Road type	8.8	27.820 (9)	<.001	88.4	91.5
3.	Road layout	14.6	48.650 (36)	.078	33.3	49.5
4.	Car parking	37.5	140.807 (36)	<.001	30.3	56.8
5.	Recreational space	4.0	12.483 (9)	.187	56.8	68.0
6.	View of street	15.9	53.571 (9)	<.001	53.2	70.6
7.	Natural elements in public space	4.5	14.423 (9)	.108	62.7	74.8

*Note.* REAT 2.0 = Revised Residential Environment Assessment Tool; DMA = digital map analysis.

a The numbers refer to the items of the full REAT 2.0 audit instrument (see Online Appendix B).

Four discriminant functions explained 71.9% of the variance in housing type, improving the classification accuracy to 69.4% as compared with 30.2% by chance. The parameters of the number of terraced houses, number of semi-detached houses, number of detached houses, average plot size, road angle, road width, and average distance to road significantly discriminated between the different housing type categories. Four discriminant functions explained 37.5% of the variance in car parking, of which only the first two were significant, and improved the classification accuracy from 30.3% to 56.8%. The number of terraced houses, the number of semi-detached houses, average distance to road, and average plot size significantly discriminated between the different ways cars are parked within the postcodes. One discriminant function explained 15.9% of the variance in “view of the street.” The number of detached houses, number of semi-detached houses, average distance to road, and aspect ratio with the road could significantly improve the classification accuracy from 53.2% to 74.8%. One discriminant function discriminated between different road types, accounting for 8.8% of the variance. Size of natural area within the postcode, road width, and the number of detached houses significantly discriminated between B and C roads, improving the classification accuracy from 88.4% to 91.5%.

Table 4 shows that road layout, recreational space, and natural elements could not be predicted from the nine DMA-generated urban form indicators, although separate analyses could predict the classification of the five individual natural elements at the neighborhood level (not reported here).

### Construct Validity: Neighborhood Perceptions

The construct validity of the six core components of REAT 2.0 was determined by examining its associations with residents’ perceptions of their neighborhood through survey responses. Table 5 shows the results of a series of multilevel regression models. The models included the six REAT 2.0 components as the independent variables at the unit postcode level, and the associated neighborhood perception scales as the dependent variables. The table shows the unstandardized regression coefficients (*b*), both controlled and not controlled for gender, age, and tenure status. All neighborhood perception scales were normalized by calculating the *Z* scores and the six core REAT 2.0 components recoded to a scale ranging from 0 to 1 to be able to compare the different effect sizes. The coefficients, therefore, reflect the number of standard deviations difference in neighborhood perceptions between the highest and lowest possible score for the REAT 2.0 components.

**Table 5.** Construct Validity: Unadjusted and Adjusted Associations of REAT 2.0 With Residents’ Neighborhood Perceptions.

Component code	Component description	Perception construct	Unadjusted *b* (*SE*)	Adjusted^a^ *b* (*SE*)
C1	Neighborhood condition (SL)	Perceived neighborhood condition	1.613 (0.580)**	1.781 (0.606)**
C2	Natural surveillance (SL)	Perceived natural surveillance	0.390 (0.162)*	0.440 (0.158)**
C3	Natural elements (SL)	Perceived natural elements	0.739 (0.319)*	0.685 (0.326)*
C4	Neighborhood condition (PL)	Perceived house condition	1.492 (0.558)**	1.935 (0.672)**
C5	Natural surveillance (PL)	Perceived natural surveillance	0.900 (0.310)**	0.842 (0.307)**
C6	Natural elements (PL)	Perceived natural elements	0.268 (0.458), ns	0.247 (0.467), ns

*Note.* REAT 2.0 = Revised Residential Environment Assessment Tool; *b* = unstandardized regression coefficient; SL = street level; PL = property level.

a Adjusted for gender, age, and tenure status.

* *p* < .05. **p < .01.

As expected, residents living in neighborhoods with better street-level neighborhood condition perceived fewer incivilities and maintenance issues in their neighborhood (“perceived neighborhood condition”), *b* = 1.61, 95% CI = [0.48, 2.75], and residents living in areas with better property-level neighborhood condition reported better house conditions (“perceived house conditions”), *b* = 1.49, 95% CI = [0.40, 2.59]. Better natural surveillance at both the street level, *b* = 0.39, 95% CI = [0.07, 0.71], and the property level, *b* = 0.90, 95% CI = [0.29, 1.51], contributed to better perceived natural surveillance. Neighborhoods with more natural elements at the street level were more likely to be perceived as “green” by residents (“perceived natural elements”), *b* = 0.74, 95% CI = [0.11, 1.36]. However, neighborhoods with more natural elements at the property level (i.e., trees in front garden and purposively planted vegetation) were not perceived to be greener, *b* = 0.27, 95% CI = [−0.63, 1.17]. All significant associations remained significant after adjusting for gender, age, and tenure status.

### Predictive Validity: Neighborhood Attachment

The predictive validity of REAT 2.0 was determined by examining the associations between the overall REAT 2.0 score and its six core components, with neighborhood attachment as determined by the neighborhood perception survey. Table 6 shows the regression coefficients unadjusted and adjusted for gender, age, and tenure status. The neighborhood attachment variable was normalized by calculating the *Z* scores and the REAT 2.0 components recoded to a scale ranging from 0 to 1. Residents living in neighborhoods with higher overall REAT 2.0 scores had higher levels of neighborhood attachment, *b* = 1.95, 95% CI = [0.57, 3.32]. Although it was hypothesized that each of the six REAT 2.0 components would be associated with neighborhood attachment, the study found that only property-level neighborhood condition, *b* = 2.23, 95% CI = [0.84, 3.63], and property-level natural elements, *b* = 1.07, 95% CI = [0.07, 2.07], were linked to neighborhood attachment. These associations remained significant after adjusting for gender, age, and tenure status.

**Table 6.** Predictive Validity: Unadjusted and Adjusted Associations of REAT 2.0 With Neighborhood Attachment.

Component code	Component description	Unadjusted *b* (*SE*)	Adjusted^a^ *b* (*SE*)
C1	Neighborhood condition (SL)	0.603 (0.755), ns	0.711 (0.745), ns
C2	Natural surveillance (SL)	0.384 (0.205), ns	0.383 (0.208), ns
C3	Natural elements (SL)	0.415 (0.446), ns	0.521 (0.449), ns
C4	Neighborhood condition (PL)	2.231 (0.712)**	2.427 (0.731)***
C5	Natural surveillance (PL)	0.270 (0.419), ns	0.313 (0.417), ns
C6	Natural elements (PL)	1.067 (0.510)*	1.136 (0.519)*
	Overall REAT 2.0 score	2.171 (0.779)**	2.370 (0.817)**

*Note.* REAT 2.0 = Revised Residential Environment Assessment Tool; *b* = unstandardized regression coefficient; SL = street level; PL = property level.

a Adjusted for gender, age, and tenure status.

* *p* < .05. **p < .01. ***p < .001.

## Discussion

This article described the validation of the REAT 2.0 through a series of interrelated studies and an urban modeling exercise. The instrument was validated by comparing the scores of independent observers (IRR) against a different neighborhood audit tool and digitally analyzed maps (convergent validity) and against residents’ perceptions of their own neighborhood environment (construct validity). REAT 2.0 was subsequently used to examine the role of the quality of the neighborhood environment in the emotional bonds people have with their neighborhood, as reflected in neighborhood attachment. This was also considered a test of the predictive validity of the instrument, based on the premise that good quality environments afford the development of stronger emotional bonds (although bonds may also contribute to good quality environments, as discussed later).

The research demonstrated that the REAT 2.0 has excellent IRR for individual items as well as for the six constituent components. The lowest IRR (.77 for the presence of a neighborhood watch signs) is still considered more than sufficient for most purposes (Lance, Butts, & Michels, 2006). This constitutes an improvement of the revised tool and compares well with other tools within the field. It is important that observers have the same understanding of the categories and rating scales for the assessments to be used for meaningful analyses, such as establishing the association between neighborhood quality and attachment. The results confirm that the items are clear and unambiguous, and that observers were adequately supported by a training session and user manual, leaving less space for individual biases.

The current study provided mixed results regarding the convergent validity of REAT 2.0. Most neighborhood audit tools focus on a limited number of urban form and neighborhood quality domains (e.g., Nickelson et al., 2013). For the reported results to be useful and comparable, these instruments need to capture the same aspects of the neighborhood environment through observation. This study showed that although the neighborhood condition components of REAT 2.0 corresponded with similar aspects of the PIN3 observation tool, DMA appeared to be a better way of validating some of the miscellaneous urban form elements. The classification of housing type, car parking, and view of the street could be improved by a small number of urban form parameters derived through DMA, although road layout, recreational space, and natural elements could still not be validated in this way.

The construct validity of the REAT 2.0 was demonstrated by showing that the dimension scores are associated with residents’ own perceptions of their neighborhood. In contrast to Dunstan et al. (2005), who used single items for their validation of elements of the original REAT instrument, the current study used internally consistent scales. In particular the street-level and property-level neighborhood condition components were strongly linked to perceived neighborhood condition and perceived house condition, respectively. This suggests that REAT 2.0 is based on valid neighborhood constructs to determine neighborhood quality, with two notable exceptions. First, and unlike the findings from Dunstan et al. (2005), neighborhoods with more natural elements at the property level (i.e., trees in front gardens and purposively planted vegetation in front gardens) were not perceived to be greener by its residents. This mismatch needs to be investigated in more detail in the future. It is possible that the phrasing of the green spaces questions may have led respondents to only consider the public realm. Research in the area has not been very precise in classifying the different types of greenness in the urban environment and suggests that it is necessary to make a clearer distinction between private and public natural elements (Leslie, Sugiyama, Ierodiaconou, & Kremer, 2010).

The research further found that neighborhood attachment was significantly associated with the overall quality of the neighborhood environment. However, it appeared that the association could be attributed mainly to the property-level neighborhood condition and natural elements components. This means that the study could not confirm the findings of previous research and that street-level natural elements and neighborhood condition (i.e., absence of incivilities) are important for the emotional bonds residents have with their neighborhood (e.g., Bonaiuto et al., 2003; Brown et al., 2003). Although a number of studies have shown clear relationships between the perception of green space and neighborhood attachment, the links between property-level characteristics on one hand and neighborhood attachment on the other has, to our knowledge, not been shown before. This confirms that it is important to consider both the public and private realm when making neighborhood quality assessments.

The research presented in this article has a number of strengths and limitations. A major strength is the breadth of analyses conducted to determine the validity and reliability of the REAT 2.0 tool. Not only was the IRR determined through independent observer assessments but also different elements of the REAT 2.0 were extensively validated through comparing its scores with another neighborhood assessment tool, DMA, and residents’ perceptions of the neighborhood. This means that we can have more confidence in the validity of the results regarding the links between neighborhood quality and attachment. Although the REAT 2.0 was found to be a reliable, easy-to-use instrument and most underlying constructs could be validated using residents’ perceptions of their neighborhood, the convergent validity of the miscellaneous urban form elements was less than satisfactory. The study only used a single audit instrument to assess the convergent validity of the urban form items. Future research could use multiple audit instruments to make a more comprehensive assessment of the convergent validity of the developed REAT 2.0 instrument. However, it has to be kept in mind that these urban form measures do not form part of the neighborhood quality score but are used to characterize the neighborhoods under assessment. Furthermore, other areas of research have similarly found that the convergent validity of categorical items tends to be lower than for dimensional ratings (e.g., Antony & Barlow, 2011; Renn et al., 2008). A particular difficulty of assessing the convergent validity of categorical items is that audit tools rarely use the same classification system. As the specific categories do not completely overlap, it may then not be surprising that the agreement scores are lower than for broader dimensional rating.

The DMA has shown promise in establishing convergent validity of the urban form items of REAT 2.0. However, even if the objectively derived geographic information system (GIS) measures could predict some of the urban form classifications, it left much of the variance of most urban form items unexplained. Previous research found mixed results regarding the validation of GIS-derived urban form elements with other forms of data (Duncan, Aldstadt, Whalen, Melly, & Gortmaker, 2011; Lee & Talen, 2014; Leslie et al., 2010). More work is needed to improve pattern recognition techniques to establish urban form before it can replace direct observations using neighborhood assessment tools.

The research further benefitted from the independent neighborhood assessments and attachment responses, ensuring that the results are not affected by common method variance. Despite deriving the neighborhood assessments and the attachment responses from different instruments, the study remained cross sectional; causality still cannot be inferred from the results. It is possible, and even likely, that care and maintenance of private properties and gardens are a *reflection* of neighborhood attachment as well as a *source*. Residents have more control over property-level elements than over public spaces. Pride in, or general attachment to, the neighborhood is therefore more easily expressed in the private space. The reciprocal nature of human–environment interactions should be investigated in more detail and also whether and how investments at the property level can be used to revitalize neighborhoods. Better upkeep may signal that residents care about their neighborhood and as a result may improve the reputation of the neighborhood (Wilson & Kelling, 1982), which subsequently can reduce incivilities in the public space (cf. Foster et al., 2011). The branding or reputation of the neighborhood and the role of resident’s actions therein are as yet not well understood (Bonaiuto & Alves, 2012) and would warrant further investigation.

A recurring issue in human–environment studies, including public health research, is the discussion as to what constitutes a neighborhood (e.g., Flowerdew, Manley, & Sabel, 2008; Stafford, Duke-Williams, & Shelton, 2008). Although this study used postcodes as a proxy for the neighborhood environment, they do not necessarily overlap with what residents perceive as their neighborhood. Indeed, there are indications that “neighborhood” is a fluid concept that differs from person to person and across contexts (Coulton, Jennings, & Chan, 2013). Furthermore, the term *neighborhood* was not explicitly defined in the neighborhood perception study, making it difficult to establish what scale residents used for responding to the survey. However, despite the term *neighborhood* not being explicitly defined for the residents, the study found clear associations between the independently assessed aspects of the neighborhood environment and residents’ perceptions. Previous research has shown neighborhoods are perceived to be relatively small by its residents, with perceived size being inversely related to population density (Coulton et al., 2013). Postcodes are an appropriate administrative unit to use because they are relatively small, often containing only 15 homes. However, a drawback is that their physical sizes differ. There is a risk that neighborhood quality observations are not independent from the size of the postcode, for example, there may be a greater chance of encountering incivilities in larger postcodes. Other studies have used standard-sized road segments as a unit of observation to prevent this problem from occurring (see, for example, Brownson et al., 2009).

Other limitations of the study relate to the relatively small sample size of the neighborhood perception survey, with clustering further diminishing the statistical power of the study (Snijders & Bosker, 2012). Despite its limited statistical power, the study found that nearly all REAT 2.0 components could be validated with residents’ perceptions of their neighborhood (only the property-level natural elements component could not be validated in this way).

A further limitation of the study is that it only covered a small number of neighborhood aspects. In a comprehensive review, Nickelson et al. (2013) identified 20 major and 291 subdomains measured across all assessed neighborhood audit tools. The current study was only able to assess a fraction of those domains. It has to be considered though that it is impractical and uneconomical to try to cover all domains. Indeed, most instruments only assess a subset of these domains. Modularizing an audit instrument can make it more versatile as a platform on which future research can build. The audit instrument can be customized by selecting the elements and domains that are the most relevant to the research.

## Conclusion

This study presented the results of an extensive validation exercise of the REAT 2.0 using a number of different methods and techniques, including comparisons with other neighborhood assessment tools, DMAs, and a neighborhood perception survey. The study found that the REAT 2.0 is a reliable, easy-to-use instrument to assess the overall quality of neighborhood environments. It further found that urban form features were more difficult to validate than measures of neighborhood quality. In contrast to the expectations, street-level components were not associated with neighborhood attachment. The observation that the property-level territorial functioning and natural elements components are important correlates of neighborhood attachment suggests that it may be worthwhile to further explore the role of private spaces in the outlook and revitalization of the neighborhood environment.

## Supplementary Material

Supplementary material

## Notes

1. The paper version of the Pregnancy, Infection, and Nutrition (PIN3) neighborhood audit instrument can be found in an additional file to the Evenson et al. (2009) publication: http://www.biomedcentral.com/content/supplementary/1479-5868-6-44-S1.pdf.

2. The factor loadings can be obtained from the corresponding author upon request.
