# Supplementary material for: Neighborhood Quality and Attachment: Validation of the Revised Residential Environment Assessment Tool
Source: Environ Behav. 2016 Jul 27;49(3):255–82. doi: 10.1177/0013916516634403 (PMC5316962; doi:10.1177/0013916516634403)
Supplement: Supplementary material [file EAB634403_Supplemental_materials.pdf]

Appendix A: Items and score loadings used to calculate the components and overall REAT 2.0 scores

Dimension	Street level: Item number and description[1]	Street level: Category	Street level: Loading	Property level: Item number and description[1]	Property level: Category	Property level: Loading
Neighborhood Condition	**8. Litter in public space**	No litter or refuse	**1.00**	**16. Property maintenance**	Well	**1.00**
		Predominantly free of litter and refuse	**0.67**		Minor damage	**0.67**
		Widespread distribution of litter and refuse	**0.33**		Moderate	**0.33**
		Heavily littered	**0.00**		In need of repair/desperate need of repair	**0.00**
	**9. Condition of public space**	Excellent	**1.00**	**17. Garden maintenance**	Tended fronts	**1.0**
		Good	**0.67**		Slightly neglected fronts	**0.5**
		Mixed	**0.33**		Significantly neglected fronts	**0.0**
		Poor or very poor	**0.00**			
	**10. Vandalism/graffiti in public space**	None	**1.00**	**18. External beautification**	Yes	**1**
		Some	**0.67**		No	**0**
		Moderate	**0.33**			
		Extensive	**0.00**			
Natural Surveillance	**6. View of the street**	Yes	**1**	**13. View of windows and doors**	CAN be seen clearly	**0**
		No	**0**		CAN NOT be seen clearly	**1**
Natural Elements	**7. Natural elements in public space**	Tree lined	**0.2**	**14. Trees in front gardens**	Yes	**1**
		Other trees	**0.2**		No	**0**
		Vegetation	**0.2**		Can't tell	**0**
		View natural environment	**0.2**	**15. Purposively planted vegetation in front gardens**	Yes	**1**
					No	**0**
		View park	**0.2**		Can't tell	**0**

Note: [1] the item number in the table refer to the items of the full REAT 2.0 audit instrument (see online Appendix B).

This appendix provides a short description of how the six core components and overall REAT 2.0 scores are calculated from the street-level and property-level observations. The table in this appendix shows the score loadings that were given to the different rating categories. The overall REAT 2.0 score is calculated by adding the scores of the six core components, creating a scale ranging from 0 to 10. Components were coded in such a way that higher scores represent a greater presence of the construct.

The street-level items are used to make general observations of the postcode. The *street-level neighborhood condition* component is calculated by adding together the score loadings for each item contained within it. This includes items 8 (litter in public space), 6 (condition of public space), and 10 (vandalism/graffiti in public space). Together, the three items form a scale from 0 to 3. [1] The *street-level natural surveillance* component reflects the score assigned to item 6 (view of the street), which is scored either 0 or 1 according to whether a clear view is present or not. The *street-level natural elements* component is calculated by counting the number of natural elements present in the postcode (item 7). Each natural element (such as vegetation, or a view of a park or natural environment) receives a loading of 0.2, creating a scale ranging from 0 to 1.

The property-level items require the observers to count the number of properties exhibiting a certain feature, so that each postcode under assessment will have a total number of properties

[1] The score for the property maintenance item is calculated as follows: (1.00 × number of properties with '*well'* maintained ratings + 0.67 × number of properties with '*minor* damage' ratings + 0.33 × number of properties with '*moderate'* ratings + 0.00 × number of properties with '*in need of repair*' or '*in desperate need of repair*' ratings)/number of properties in the postcode. The garden maintenance score is calculated similarly: (1.00 × number of gardens with '*tended fronts*' ratings + 0.5 × number of gardens with '*slightly neglected*' ratings + 0.00 × number of gardens with '*significantly neglected*' ratings)/number of front gardens in postcode. The external beautification score simply reflects the proportion of properties within the postcode that have some sort of external beautification.

for each item (e.g., X properties with and Y without external beautification). Each item is converted into a scale ranging from 0 to 1 using the loadings listed in Table 1. A score of 0 reflects that all properties or gardens received the lowest rating and a score of 1 reflects that all properties or gardens received the highest rating (although the majority of postcodes will fall somewhere in between). The overall score for the *property-level neighborhood condition* component is calculated by adding the loaded scores for all the items contained within it. This includes items 16 (property maintenance), 17 (garden maintenance), and 18 (external beautification). Together, the three items form a scale from 0 to 3. The *property-level natural surveillance* component reflects the proportion of properties within the postcode of which the ground floor windows and doors can be seen clearly from the street, forming a scale from 0 to 1. For the *property-level natural elements* component, the trees in front gardens score reflects the proportion of properties within the postcode that have trees in their front garden, and the purposively planted vegetation in front gardens score reflects the proportion of properties within the postcode that have purposively planted vegetation (other than trees) in their front garden. The ‘trees’ and ‘purposively planted vegetation’ in front gardens scores are then averaged to create a score from 0 to 1.

Appendix B: The Revised Residential Environment Assessment Tool (REAT 2.0)

**REAT 2.0 audit instrument**	Street name: ______________________________<br>Number of Properties: ______________________<br>Postcode: _________________________________<br>SOA code: _________________________________	Auditor: _____________ Date: ________________<br>Start time: ___________ Finish time: ___________

**Housing and Road Type** (Miscellaneous)	
1. Housing type	a) Detached b) Semi-detached c) Terraced d) Flats e) Mixed
2. Road type	a) A Road b) B Road c) C/local Road
3. Road layout	a) Closed cul-de-sac b) Open cul-de-sac c) No-through road d) Through road

Street Level Observations

Miscellaneous

4. How are cars mainly parked?

☐ On street, one side
☐ On street, both sides
☐ Predominantly public courts
☐ Predominantly off street private parking
☐ Mixed (on street and private)
☐ Can't tell

5. Any recreational space *(inc. non-green)* **that children could play on?**

☐ Yes
☐ No

Natural Surveillance

6. Can you get a clear view of the whole street and houses?

☐ Yes
☐ No

Natural Elements

7. Does any of the following apply? *(tick all that apply)*

☐ The road is tree lined
☐ There are other purposively planted trees in public spaces
☐ There is purposively planted vegetation in public spaces
☐ There is a view of the natural environment *(countryside, mountain, sea)*
☐ There is a view of a park/green area *(man made)*

Neighborhood Condition

8. How littered are the streets?

☐ No litter or refuse
☐ Predominantly free of litter and refuse except for some small items
☐ Widespread distribution of litter and refuse with minor accumulations
☐ Heavily littered with significant accumulations

9. What is the general condition of public spaces?

☐ Excellent *(mint condition, one minor fault)*
☐ Good *(good except minor isolated repairs)*
☐ Mixed *(mix of well and poorly maintained items)*
☐ Poor or very poor *(obvious and significant neglect)*

10. How much vandalism/graffiti is present on both public spaces and private properties?

☐ None
☐ Some *(2 or less small occurrences)*
☐ Moderate *(many small or up to one significant occurrence)*
☐ Extensive *(large areas of small or more than one significant occurrence)*

Miscellaneous

11. Any neighborhood watch signs? *(on houses or lampposts)*

☐ Yes
☐ No

Observations:

Property level observations

Miscellaneous

12. What is the nature of the space immediately outside front doors? *(Count)*

	With clear barriers impeding entry	*Without clear barriers impeding entry*
Private		
Shared with neighbour(s)		

Public (footpath/ street/ public area)___________________

Natural Surveillance

13. Can you get a clear view of ground floor windows or doors from the street?

Yes, can be CLEARLY seen

____

No, cannot be CLEARLY seen

____

Natural Elements

14. Trees in front gardens that are obvious from road?

Yes____

No____

15. Houses with purposively planted vegetation? *(including healthy pots and baskets)*

Yes____

No____

Can't tell____

Neighborhood Condition

16. How well maintained are properties from the outside? *(Count) (Look at roof, windows, doors, walls, fascias, guttering and any permanent fixtures/fittings in front garden/yard)* (*specify if any property is not residential*)

Well *(mint condition)*

____

Maintained with minor faults *(few, small and easy DIY repairs)*

____

Moderate *(more substantial cosmetic DIY repairs)*

____

In need of repair *(Structural attention)*

____

In desperate need of repair *(extensive refurbishment) (include any clearly derelict or vacant property and land)*

____

Can't tell____

17. How well cared for are properties' front gardens or spaces?

Tended fronts *(cared for regularly)*

____

Slightly neglected/ indifferent *(slightly overgrown, small items of litter, no signs of anything)*

____

Significantly neglected and/or littered *(significantly overgrown, considerable litter)*

____

No fronts____

Can't tell____

18. Properties with some sort of external beautification? *(pots, garden furniture, decorative items)*

Yes____

No____

Can't tell____

Observations:
